# A Large Skull Defect Due to Gorham-Stout Disease: Case Report and Literature Review on Pathogenesis, Diagnosis, and Treatment

**DOI:** 10.3389/fendo.2020.00037

**Published:** 2020-02-05

**Authors:** Catherine E. de Keyser, Michael S. Saltzherr, Eelke M. Bos, M. Carola Zillikens

**Affiliations:** ^1^Department of Internal Medicine, Bone Center, Erasmus University Medical Center, Rotterdam, Netherlands; ^2^Department of Radiology and Nuclear Medicine, Erasmus University Medical Center, Rotterdam, Netherlands; ^3^Department of Neurosurgery, Erasmus University Medical Center, Rotterdam, Netherlands

**Keywords:** Gorham-Stout, osteolysis, rare bone disease, parietal bone, bone graft

## Abstract

A 24-year old man was referred to the Erasmus MC Bone Center because of an asymptomatic increasing skull defect of the left parietal bone. The defect was first noticed at the age of six, and gradually increased over the years. His medical history was unremarkable, without any known trauma and a negative family history for bone diseases. Laboratory tests showed a low vitamin D level without other abnormalities. Particularly, there was no increase in markers of inflammation or bone turnover. CT-scans of the skull showed an osteolytic region of the parietal skull bone, with a two-centimeter increase in diameter over 9 years. Contrast enhanced MRI showed lymphangiogenic invasion, which was compatible with our suspicion of Gorham-Stout disease. The patient was referred to the neurosurgeon for treatment with a bone graft while considering additional drug treatment. Gorham-Stout or vanishing bone disease is a rare entity characterized by progressive osteolysis with lymphangiogenic bone invasion. Although already reported in 1838, currently the diagnosis and treatment of Gorham-Stout disease is still challenging. The underlying pathophysiology is not clarified yet and several theories exist. The disease usually affects persons younger than 40 years and the majority present with bone disease of the maxillofacial region, the upper extremities or the torso. The clinical presentation includes most frequently pain, swelling, and functional impairment of the affected region, but the disease can also be asymptomatic. Laboratory investigations are usually normal, and diagnosis is based upon imaging and sometimes pathology examination of affected bone tissue. Treatment is experimental and there is no general consensus about the best option due to lack of randomized controlled trials. Case reports showed patients treated with bisphosphonates, interferon-alpha, anti-VEGF therapy, mTOR inhibitors, and radiotherapy. There are some reports of surgery with prosthetic or bone grafts but no long-term follow-up data exist. This paper describes a unique case of Gorham-Stout disease of the parietal skull bone and discusses the current state of knowledge about this rare bone disease.

## Case Presentation

A 24-year old man was referred to the Erasmus MC Bone Center in Rotterdam, the Netherlands, because of a growing skull defect of the left parietal bone. He had been analyzed in the referring hospital because the defect became larger over time, but no treatment was initiated. The defect was first noticed when he was 6 years old, and he nor his parents could remember any traumatic incident. His medical history mentioned no relevant diseases and he did not use any medication. He reached his target height with no other skeletal deformities, had no other complaints and was in good clinical condition. Family history was negative for bone diseases.

**Figure 1:**
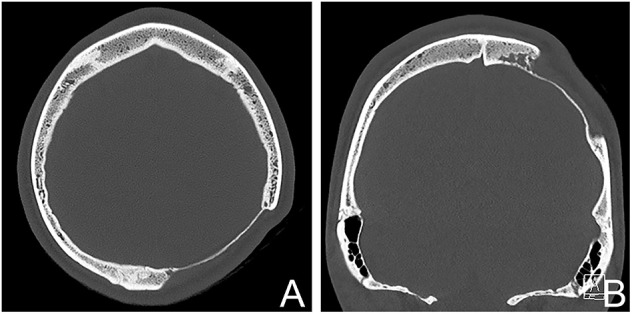
CT-scan of the patient showing the osteolytic skull defect of 60 mm. **(A)** Axial plane, **(B)** coronal reconstruction showing irregular lobular vessel shaped osteolysis at the medial side.

**Figure 2:**
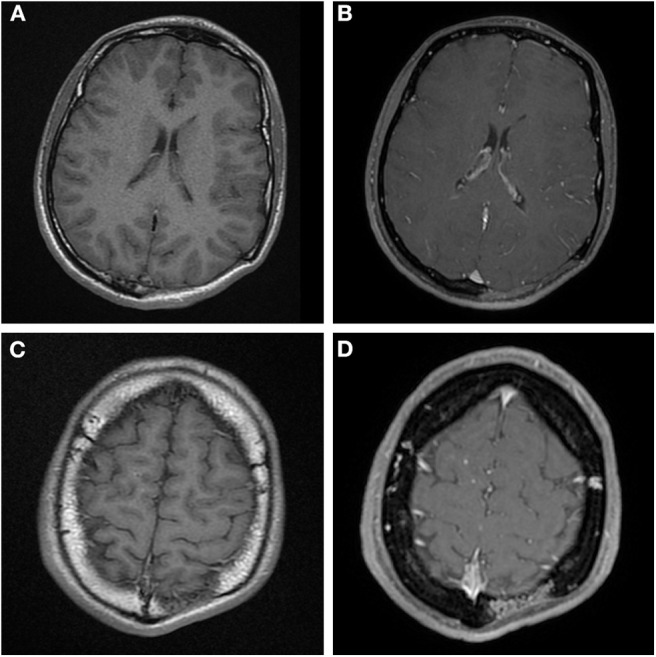
**(A,C)** Axial T1 MR images before contrast administration, **(B,D)** post contrast T1 weighted MR images at the same level as **(A,C)**. The main area of osteolysis **(A,B)** is filled with non-enhancing soft-tissue. But there is vascular shaped contrast enhancement in the diploë in **(D)** at the edge of the osteolysis, this is the same area as mentioned in Figure 1B.

**Figure 3:**
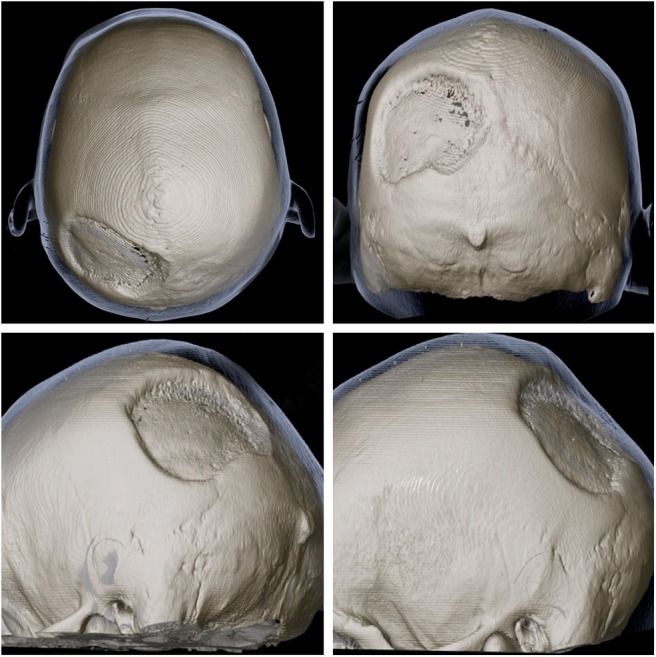
3D reconstruction of skull CT from multiple angles showing the extensiveness and location of the defect of the left parietal bone.

Laboratory tests showed a low 25-hydroxy vitamin D level (21 nmol/L, reference values 50–120 nmol/L), no increase in inflammation markers [C-reactive protein (CRP), erythrocyte sedimentation rate (ESR)], and normal bone turnover markers in the form of alkaline phosphatase, procollagen type 1 N propeptide (P.1.N.P.), and beta isomer of C-terminal telopeptide of type 1 collagen (beta-CTX) with only slightly increased bone alkaline phosphatase (30.0 μg/L, reference value <20.1 μg/L). Also, serum levels of cytokines that may be involved in the pathogenesis [interleukin-6 (IL-6), tumor necrosis factor alpha (TNF-α), interleukin-1-beta (IL-1β)], were normal. The results of the most relevant laboratory tests are shown in Table 1. Laboratory test were performed according to standard procedures. CT-scans of the referring hospital showed a region of osteolysis of the diploë and outer table of the parietal bone, with an intact inner table. The region size of the osteolytic region slowly increased in size over the years. The first CT-scan was performed at the age of 15 and showed a defect with a maximum diameter of 38 mm. One year later the defect had increased to 41 mm. CT-scans at the age of 22 and 24 showed an increase of the defect to a maximum diameter of 57 and 60 mm, respectively. Figure 1 shows the most recently performed CT-scan from the referring hospital with the defect of 60 mm. 3D-CT reconstructions were made to visualize the extensiveness of the defect (Figure 2). Based on the clinical manifestation and radiological findings the diagnosis Gorham-Stout disease was suspected. To confirm this diagnosis, we performed a contrast enhanced MRI-scan which showed an enhancing zone of diploic vascularity at the edge of the osteolytic region (Figure 3), characteristic of Gorham-Stout. We therefore concluded that our patient suffered from Gorham-Stout disease of the parietal bone. Additional bone scintigraphy showed no other lesions. We were challenged by the decision to cover the defect with or without removal of affected bone and whether or not to start additional medical treatment. Due to the rarity of the disease, lack of literature with respect to the underlying pathophysiological mechanism and a standardized treatment guideline, we performed a literature search to choose the best clinical approach and also consulted experts in the field.

**Table 1:** Results of the laboratory tests from the patient.

**Laboratory test**	**Serum value (reference values)**	**Laboratory test**	**Serum value (reference values)**
Calcium	2.51 (2.20–2.65 mmol/L)	CRP	<0.3 (<10 mg/L)
Phosphate	1.04 (0.80–1.40 mmol/L)	ESR	2 (0–15 mm/h)
Albumin	53 (35–50 g/L)	ALP	84 (<115 U/L)
25-OH-D	21 (50–120 mmol/L)	Gamma-GT	23 (<55 U/L)
IL-6	<10 (<10 pg/mL)	P.1.N.P.	93 (19.4–95.5 μg/L)
TNF-α	<15 (<15 pg/mL)	Beta-CTX	0.27 μg/L
IL-1β	<10 (<10 pg/mL)	Bone AF	30.0 (<20.1 μg/L)

*IL-6, interleukin 6; TNF-α, tumor necrosis factor alpha; IL-1β, interleukin-1-beta; CRP, C-reactive protein; ESR, erythrocyte sedimentation rate; ALP, alkaline phosphatase; Gamma-GT, gamma glutamyltransferase; P.1.N.P., beta isomer of C-terminal telopeptide of type 1 collagen; beta-CTX, beta isomer of C-terminal telopeptide of type 1 collagen*.

## Overview of the Literature on Gorham-Stout Disease

Gorham-Stout disease, also named vanishing bone disease, phantom bone disease, or idiopathic massive osteolysis, is a rare bone disorder characterized by progressive osteolysis with lymphatic and vascular proliferation (1). The incidence is very rare, with only a few hundred case reports described in literature (1, 2). The disorder does not seem to exhibit a preference for race, geographic area, or sex, although the disease might be slightly more prevalent in males. No definite pattern of genetic inheritance has been described, currently no cases with a family history of the disease have been reported. It can be diagnosed at every age, although most of the cases that have been described occurred under the age of 40 years, with an average age at diagnosis of 25 years (1, 3, 4).

### Clinical Picture

Gorham-Stout disease most frequently involves the upper part of the body. Most cases present with affected bones of the maxillofacial region, vertebrae, ribs and the pelvic girdle, but it can affect any part of the skeleton. In the majority of the cases the disease is monostotic, involvement of multiple bones has been described (3). There is no clear trigger in clinical practice that causes the disease to occur. Sometimes the disease can occur after a traumatic incident. After a trauma, the disease is confined to a single location (5).

The clinical presentation and patient's complaints are dependent on the bone that is affected. Patients most frequently present with local pain. This can be accompanied by functional impairment, muscle weakness or local edema. The disease can also be completely asymptomatic, or may have a first presentation with a spontaneous or traumatic fracture. Dyspnea can occur due to chylothorax, a frequently seen complication in Gorham-Stout disease of the bones in the thorax with a prevalence up to 25%. Pleural effusion and chylothorax may develop when the lymphoangiogenic invasion extends to the pleural cavity or the thoracic duct (6).

The prognosis of Gorham-Stout disease is unpredictable and varying, based on the extent, severity, and localization of the disease. It is considered a benign disease and its natural progression is characterized by spontaneous resolution. When the disease has stabilized, the resorbed bone is not replaced by new bone. Severe complications have been described and can be life threatening. Besides the above described chylothorax due to pleural involvement, rarely described complications include hemangiomatous lesions of the skin, pericardial effusion, osteomyelitis with septic shock, increased intracranial pressure, meningitis, cerebrospinal fluid leakage, spinal cord involvement and paraplegia by vertebral involvement (4, 7–9). In general, visceral and spinal involvement are associated with a poor prognosis (4).

### Diagnosis

It can be a challenge to diagnose patients with Gorham-Stout disease on the clinical, histological, and radiological features. The disease can be misdiagnosed since it resembles other clinical diseases that also present with pain of the musculoskeletal tract, including rheumatoid arthritis, osteoarthritis, or osteomyelitis, and other causes of osteolysis such as intraosseous malignancies, granulomatous diseases, or central nervous system diseases such as a meningioma or syringomyelia. In clinical practice, it is often a diagnosis by exclusion, after ruling out inflammatory, infectious, metabolic and neoplastic diseases (10). There is no standardized guideline for diagnosis. Heffez et al. proposed an algorithm with eight diagnostic criteria for Gorham-Stout disease. These are based on clinical, radiological and histopathological findings and are the following: (1) angiomatous tissue present in biopsy tissue, (2) absence of cellular atypia, (3) minimal or no osteoblastic response and absence of dystrophic calcifications, (4) evidence of local progressive osseous resorption, (5) non-expansive, non-ulcerative lesion, (6) absence of visceral involvement, (7) osteolytic radiographic pattern, (8) negative hereditary, metabolic, neoplastic, immunologic, and infectious etiology (11).

Laboratory tests are generally not helpful in the diagnosis of Gorham-Stout disease since they are usually normal. There is currently no specific biomarker that can be used to assess the disease severity or that can be used in the follow-up to monitor the response to therapy. Alkaline phosphatase may be increased but is frequently normal (4, 10). Authors from a recent case report on Gorham-Stout disease with vanishing of three ribs and an osteolytic lesion of the left humerus bone suggest an initial set of laboratory tests to use in the diagnostic phase, including bone turnover markers. Since in this case, the bone turnover markers P.1.N.P. and bone-specific alkaline phosphatase were increased, the authors propose to use the bone turnover markers also for follow-up (12). In reported studies, several factors were measured in the blood of patients with Gorham-Stout disease (e.g., vascular endothelial growth factors, IL-6, sRANKL, and osteoprotegerin). IL-6 and VEGF-A were elevated in the blood of patients with Gorham-Stout disease (3), and in a recent study also the levels of pyridinoline cross-linked carboxyterminal telopeptide of type I (ICTP) and sclerostin (13). However, they can also be normal.

Conventional X-rays are not useful for early diagnosis of the disease. Radiology findings at an early stage resemble patchy osteoporosis and show radiolucent foci at intramedullary and subcortical zones. If the disease progresses, bone deformities develop with bone mass loss, atrophy, fracture, fragmentation, and finally the image of vanishing bone (10, 14). Bone scintigraphy mostly shows increased uptake in the blood pool phase at the region of increased lymphatic and vascular proliferation, and a region of decreased uptake at the osteolytic region of vanished bone (4, 10, 15, 16). CT-scan is useful to investigate the location and extent of the osteolytic bone, and can sometimes show vessel shaped defects at the edge of osteolysis, as in our case, but is mostly not characteristic for diagnosis. MR imaging also shows the osteolysis and can clearly show the vascular and/or lymphatic vessels within the bone which enhance after contrast administration at the region of active osteolysis (17). Finally, the diagnosis can be confirmed by histology from a biopsy of the affected bone. In our patient, no biopsy of the affected region was taken, as the MR characteristics of an osteolytic region filled with non-enhancing soft-tissue and vascular shaped contrast enhancement in the diploë at the edge of the osteolysis, were very characteristic for the disease, and there was no clinical or laboratory suspicion for an alternative inflammatory, neoplastic, or infectious cause of the osteolysis. Recently, a multidisciplinary guideline for the initial evaluation of complicated lymphatic anomalies including Gorham-Stout disease was published (18). Although based on expert opinion consensus, authors present a diagnostic approach including laboratory, radiological, and histological evaluation. They state that the need for a histological biopsy should be discussed in a multidisciplinary expert team after the clinical and radiological evaluation. A possible complication of a biopsy is the oozing of lymphatic fluid from the biopsy site, which may need lymph drainage or can induce a chylothorax in the case of a biopsy of an affected rib (18).

### Pathogenesis

The first case of Gorham-Stout disease was already reported in 1838 and described a patient with a vanishing of the complete humerus bone over 11 years (19). After more than a century, in 1955, Gorham and Stout investigated the characteristic histopathological findings of the massive osteolysis based on 8 cases. They discovered that the massive osteolysis was accompanied by angiogenesis and lymphangiomatosis, with proliferation of benign vascular structures and destruction of osseous matrix. The lymphangiomatosis resulted in a zone of hyperemia, which disrupts normal bone metabolism and shifts the bone metabolism to favor osteoclastic activity. The destructed bone tissue is replaced by fibrous tissue and no new bone formation occurs (20). Further research with the development of immunohistochemical markers of lymphatic endothelial cells revealed that in Gorham-Stout disease lymphatic anomalies are present in cortical and medullary parts of the bone, and that these lymphatic vessels are normally not present in bone tissue (3, 21). Although these findings were discovered decades ago, currently the exact pathophysiologic mechanism is not clarified. The proliferation of lymphatic vessels plays a central role in the pathogenesis of the disease and several mechanisms appear to be involved in the process of osteolysis.

An important role is attributed to the activation of osteoclasts through the immune system. In Gorham-Stout disease, an imbalance exists between osteoblasts and osteoclasts, favoring osteoclast activity leading to bone resorption over bone formation. Studies showed that *in vitro*, the osteoclast differentiation is increased, while the function of the osteoblasts is impaired with decreased bone mineralization ability (13). Osteoclasts are activated by different cytokines which are secreted by cells from the immune system. T-lymphocytes produce cytokines such as interferon-gamma, tumor necrosis factor alpha, prostaglandin E2, and interleukin 17, which induce the cytokine receptor activator of nuclear factor kappa-B ligand (RANKL). RANKL binds to the RANK receptor expressed on osteoclast precursors, leading to differentiation and activation of the osteoclasts (3). Furthermore, T-lymphocytes, but also leukocytes, osteoblasts, and dendritic cells, produce IL-6 and consequently activate mesenchymal stem cells in the bone marrow, stimulating RANKL production (22, 23). As described above, in case reports of Gorham-Stout disease the serum levels of IL-6 are sometimes elevated (3, 13, 24). Whether there is a systemic immune response, reflected by increased levels of cytokines in the serum of some patients, or whether these cytokines act locally at the defective bone, is currently not known.

Another role in the pathophysiological mechanism of the disease is attributed to macrophages, that can induce osteoclast activity and stimulate lymphangiogenesis through the production of VEGF-A, -C, and -D (13, 22, 25). Furthermore, they can inhibit osteoblast function by the production of TNF-α. The growth factors VEGF-A and VEGF-C, but also platelet-derived growth factor-BB, play a role in the invasion of lymphatic vessels (26). These growth factors signal through the same pathway which ends in the mammalian target of rapamycin (mTor) (27–29). mTor is an important kinase in the progression of the cell cycle and a key regulator of immune responses.

Furthermore, a role may be played by calcitonin, a hormone which is produced by the parafollicular (C-cells) of the thyroid gland and which has anti-osteoclastic properties. Interestingly, a patient who has been reported with agenesis of these C-Cells developed Gorham-Stout disease (30). Calcitonin binds to the calcitonin receptor on osteoclasts, thereby inhibiting their activity and thus inhibit osteoclastic bone resorption. This way, lack of calcitonin can contribute to the osteolysis in Gorham-Stout disease.

In the occurrence of Gorham-Stout disease after a trauma or fracture, it is hypothesized that the pro-inflammatory response after injury with cytokine secretion plays a role in the development of the disease. For example, secreted interleukin-1 (IL-1) induces angiogenesis and enhances the formation of cartilaginous callus, which is also a histological finding in Gorham-Stout disease.

However, to reveal the exact molecular triggers and subsequent mechanisms that cause Gorham-Stout disease, more research is needed. It would be of most interest to reveal the trigger or the origin of the lymphangiomatosis, finally leading to osteolysis. Elucidating this may lead to a possible target for therapy, which might allow early cessation of the cascade and preventing bone destruction and loss.

### Treatment

Since the underlying pathogenesis has not been clarified, a targeted treatment strategy is lacking. Furthermore, no prospective randomized controlled trials on treatment of Gorham-Stout disease have been performed due to the rarity of the disease. Several therapeutic options have been suggested to be beneficial but results appear to be variable. Generally, treatment consists of conservative therapy with medication, radiotherapy, or invasive therapy with surgery. The choice for a certain treatment is currently based on case reports from literature and expert opinion.

The pharmacological treatment options target inhibition of osteoclast activity, inhibition of angiogenesis or suppression of the immune system. Pharmacological agents that have been reported are bisphosphonates, interferon-a 2b, mTor inhibitors, vitamin D, calcium, calcitonin, anti-VEGF antibodies, bevacizumab, bleomycin, thalidomide, somatostatin, androgens, propranolol, low-molecular-weight heparin, and glucocorticosteroids (3). Patients have been treated with monotherapy, but frequently a combination of drugs has been used, such as bisphosphonates with sirolimus (27) or bisphosphonates with interferon-a-2b (31). Pharmacological treatment is also frequently combined with surgery. Bisphosphonates are thought to be beneficial through inhibition of osteoclast-mediated bone resorption, and therefore counteract the osteolysis in attempt to stabilize the disease process. Clinically, improvement of local pain and inhibition of further osteolysis has been described (31, 32). In the reported cases, bisphosphonates were mostly supplied intravenously. Interferon-a 2b and thalidomide may be beneficial through immunomodulatory and antiangiogenic effects. M-TOR inhibitors such as sirolimus and everolimus inhibit the activation of T-lymphocytes by inhibition of the intracellular signal transduction. mTOR is an important kinase in the progression of the cell cycle. The net result is immune suppression by inhibition of lymphocytes and decreased lymphangiomatous invasion by inhibition of lymphangiogenic growth factors (33, 34). The beta-blocking agent propranolol has also been reported as therapeutic option for Gorham-Stout disease, possibly through lowering of VEGF-A levels (35). In their description of 186 reported cases from literature with Gorham-Stout disease Dellinger et al. present the clinical features including affected bones and applied treatments in an overview table in the supplementary material (3).

Radiation therapy may prevent progression of bone osteolysis by inhibition of endothelial cell proliferation. It may prevent disease progression or induce disease regression in 77–80% of the Gorham-Stout disease cases. These percentages are based on a literature analysis on 44 cases with Gorham-Stout disease in which radiation therapy was used, and of results of 10 cases with Gorham-Stout disease obtained from structured questionnaires taken in radiation therapy settings, respectively. The treatment seemed safe, with a mild early and late toxicity after a dose of 30–45 Gy in total (36).

Surgical treatment is applied if patients complain about severe pain and impaired function (7, 37, 38), if there is a fracture or a high risk of fracture, or if complications occur such as an increased intracranial pressure (39) or chylothorax (40) that need surgical intervention. Surgery can consist of removal of the affected bone tissue, with subsequent reconstruction by the use of bone grafts, prostheses, or both.

Regarding the treatment of our case, lesions of the skull have rarely been described in the parietal bone. More frequently reported are skull-base defects, associated with cerebro-spinal fluid leak and meningitis (3, 41). Furthermore, involvement of the mandibulum is a frequently described location in the skull (3). There are only two other reported case reports of Gorham-Stout disease of the parietal bone. Amirjamshidi et al. described a 62-year old man with a painful and tender lesion of the right skull region, caused by a few centimeters large lytic lesion of the parietal bone with MRI characteristics of Gorham-Stout disease. Treatment with pharmacological therapy including bisphosphonates and interferon-alpha−2b did not result in pain relief. Subsequently, he underwent craniotomy with removal of all affected bone and replacement by a titanium plate. After this surgery, he was free of complaints and follow-up after 1 year showed no recurrent disease (42). Parihar et al. described a 35-year old female patient with Gorham-Stout disease of the left parietal bone of the skull, at the same location that was also painless. The defect had a diameter of about 40 mm. Treatment only consisted of surgery with replacement of the affected bone using cranioplasty with bone cement. Unfortunately, no follow-up data is available (43).

## Case Follow-Up

After the MRI-scan of the skull, we concluded that our patient suffered from Gorham-Stout disease of the parietal bone. Since his disease was progressive over the years, we attempt to determine a proper treatment strategy with the goal to protect the brain from mechanic injury locally at the defective zone, and to prevent further osteolysis. But determining the most adequate treatment approach is challenging since literature is not conclusive on the precise treatment approach, reports only a few patients with Gorham-Stout disease of the parietal bone, and currently no guideline or widely accepted treatment approach exists. We referred the patient to the neurosurgeon for surgical treatment of the defect. Since only the inner table is still present over a large area, there is an indication for cranioplasty to protect the brain against mechanical injury. Based on experience and literature from comparable bone defects of the skull with other underlying causes, there are two possible surgical approaches. First, to cover the defect with a custom made 3D printed polyetheretherketone (PEEK) graft without removing all of the affected bone. A possible complication of this treatment is progression of the osteolysis around the graft and release of the graft with the potential necessity for repeat surgery in the future. This approach is less invasive and carries less risk than the second approach, which would consist of the removal of all the affected remaining bone including a margin of surrounding healthy bone tissue and replacement with a custom made PEEK graft. This included the skull over the superior sagittal sinus. Complications of this operation are the risk of major bleeding from the venous sinuses, venous sinus thrombosis causing cerebral infarction, and -although unlikely- death. From literature, it cannot be concluded whether removal of the affected bone with a margin will prevent recurrence of disease and whether a more invasive and riskier operation should be considered to cure the patient. Importantly, no long-term follow-up data of either procedure are available.

We decided to treat our patient with the first above mentioned surgical approach in combination with an intravenously supplied bisphosphonate. Currently, a custom made PEEK-bone graft is being developed to cover the defect. During surgery we will collect tissue for histological confirmation of the diagnosis. After the surgery, we will supply zoledronic acid five milligrams intravenously with the intent to stop further osteolysis. Follow-up visits and CT and MRI-scans on a regular basis will be used to determine the duration of bisphosphonate treatment and will explain whether the allocated therapy brings the osteolysis to a halt.

## Conclusion

We describe the case of a young man presenting with a 60 mm defect in the parietal bone of the skull due to Gorham-Stout disease and discuss the challenging task to determine an appropriate treatment strategy to prevent further osteolysis and protect his brain from mechanical damage.

Gorham-Stout disease is a rare bone disease characterized by progressive osteolysis with lymphatic and vascular proliferation. Physicians should be aware of the existence of this disease and once the diagnosis is suspected, referral to a medical center dedicated to rare bone diseases is recommended. Future research is needed since the exact underlying mechanism is not revealed yet. Research should focus on the triggers that initiate and maintain the underlying pathophysiological mechanisms, to get more insight directing toward the most appropriate treatment strategy.

Our case is unique, since it is only the third patient reported in literature with Gorham-Stout disease of the parietal bone of the skull with the largest defect. Future follow-up visits are needed to show whether further osteolysis occurs.

## Ethics Statement

The patient provided written informed consent for publication of the case report.

## Author Contributions

CK wrote the first draft of the manuscript. EB, MS, and MZ contributed to the design and writing. All authors made critical revisions of the manuscript. EB provided the 3D reconstructions of the skull. MS provided the other figures. All authors read and approved the submitted and revised version.

### Conflict of Interest

The authors declare that the research was conducted in the absence of any commercial or financial relationships that could be construed as a potential conflict of interest.
